# An integrated machine learning approach for predicting DosR-regulated genes in Mycobacterium tuberculosis

**DOI:** 10.1186/1752-0509-4-37

**Published:** 2010-03-31

**Authors:** Yi Zhang, Kim A Hatch, Joanna Bacon, Lorenz Wernisch

**Affiliations:** 1 School of Crystallography, Birkbeck College, University of London, Malet Street, London, WC1E 7HX, UK; 2 TB research, Health Protection Agency, CEPR, Porton Down, Salisbury SP4 0JG, UK; 3 MRC Biostatistics Unit, University Forvie Site, Robinson Way, Cambridge CB2 0SR, UK

## Abstract

**Background:**

DosR is an important regulator of the response to stress such as limited oxygen availability in *Mycobacterium tuberculosis*. Time course gene expression data enable us to dissect this response on the gene regulatory level. The mRNA expression profile of a regulator, however, is not necessarily a direct reflection of its activity. Knowing the transcription factor activity (TFA) can be exploited to predict novel target genes regulated by the same transcription factor. Various approaches have been proposed to reconstruct TFAs from gene expression data. Most of them capture only a first-order approximation to the complex transcriptional processes by assuming linear gene responses and linear dynamics in TFA, or ignore the temporal information in data from such systems.

**Results:**

In this paper, we approach the problem of inferring dynamic hidden TFAs using Gaussian processes (GP). We are able to model dynamic TFAs and to account for both linear and nonlinear gene responses. To test the validity of the proposed approach, we reconstruct the hidden TFA of p53, a tumour suppressor activated by DNA damage, using published time course gene expression data. Our reconstructed TFA is closer to the experimentally determined profile of p53 concentration than that from the original study. We then apply the model to time course gene expression data obtained from chemostat cultures of *M. tuberculosis *under reduced oxygen availability. After estimation of the TFA of DosR based on a number of known target genes using the GP model, we predict novel DosR-regulated genes: the parameters of the model are interpreted as relevance parameters indicating an existing functional relationship between TFA and gene expression. We further improve the prediction by integrating promoter sequence information in a logistic regression model. Apart from the documented DosR-regulated genes, our prediction yields ten novel genes under direct control of DosR.

**Conclusions:**

Chemostat cultures are an ideal experimental system for controlling noise and variability when monitoring the response of bacterial organisms such as *M. tuberculosis *to finely controlled changes in culture conditions and available metabolites. Nonlinear hidden TFA dynamics of regulators can be reconstructed remarkably well with Gaussian processes from such data. Moreover, estimated parameters of the GP can be used to assess whether a gene is controlled by the reconstructed TFA or not. It is straightforward to combine these parameters with further information, such as the presence of binding motifs, to increase prediction accuracy.

## Background

Although gene microarrays enable us to measure the abundance of gene transcripts, they fail to capture any changes in transcription factor activity (TFA) after transcription, for example, during the translation process or by the interaction of the transcription factor with other proteins or cofactors. The discrepancy between the activity of a transcription factor (TF) and its expression profile may be substantial.

Consequently, TFAs might need to be modelled as hidden variables as has been suggested in several publications [1-4].

### Reconstruction of hidden transcription factor activity

Liao et al. (2003) [1] propose network component analysis (NCA) which estimates hidden TFAs based on known connections between TFs and their target genes. Sabatti and James (2006) [4] present a Bayesian latent component algorithm for transcription regulatory networks based on work by West (2003) [5]. In this Bayesian framework, priors incorporate biological knowledge of interactions between TFs and target genes. An advantage of a Bayesian approach is also the ability to identify unknown network connections using priors that encourage sparsity of the connections. Pournara and Wernisch (2007) [6] provide an overview and comparison of these and similar approaches based on factor analysis. Such approaches make no use of the order in time and the dynamics of the data. A state space model with linear dynamics is suggested in Sanguinetti et al. (2006) [7]. Pournara and Wernisch (2008) [6] extend the Bayesian factor analysis approach for reconstructing TFAs and connections by integrating correlation between time points. Once a TFA is reconstructed it can be further exploited to predict unknown target genes. For example, Barenco et al. (2006) [8] present a differential equation based model to estimate the hidden TFA of p53 and then predict the target genes of p53 using the hidden TFA derived from the model. This method assumes a log-linear relationship between a TF and its target genes.

In general we expect a target gene to respond to its regulator in a nonlinear fashion due to saturation and threshold effects (Nachman et al. (2004) [9]). So it is more realistic to assume both linear and nonlinear interactions between a TF and its target genes. Furthermore, despite using time course gene expression data, the above methods either fail to account for time continuity of a TFA or assume linear dynamics on the TFAs.

### State space model with Gaussian processes

In this study, we propose a novel strategy to estimate the hidden TFA of a regulator and to predict its target genes. This approach makes use of Gaussian processes (GP) to account for possibly nonlinear dynamics of the hidden TFA, and to model nonlinear target gene responses of regulated genes.

To reconstruct hidden TFAs from the microarray data, we applied and extended the Gaussian Process dynamic model (GPDM), which was previously used for tracking human motions [10]. In the GPDM, nonlinear state maps are used to model the dynamics of hidden TFAs and output maps reflect nonlinear regulation of the target genes. Both the state and output maps are defined by Gaussian processes. We use a modified implementation of the GPLVM MATLAB code provided by Lawrence (2005) [11]. The details of the model implementation and extensions are described in the methods section.

Intuitively, given a training set of *F*-dimensional input data points with known output values, a Gaussian process fits a smooth nonlinear interpolation surface representing this output relation as a function (strictly speaking as a family of functions weighted by a probability distribution, see the methods section). In this work, motivated by our interest the DosR regulator, we only use one input dimension, *F *= 1, but the approach can be easily generalised to more than one regulator and higher *F*. The smoothness of the interpolation, which is estimated in a training phase, is mostly guided by the "roughness" of the input data and the choice of smoothness priors.

Each of the *d *input dimensions is linked to a *relevance parameter*. If this parameter is small, the interpolated function is flat in this dimension and this particular dimension cannot exert much influence on the output. Thus relevance parameters provide a principled way to decide which genes might be under the influence of a regulator: if the relevance parameter is small the gene response is unlikely to depend on the TFA of this regulator.

We use two types of GPs in our approach: one for the dynamics and another for the output. The dynamics is modelled by a GP mapping the value of the regulator at a time point to its value at the next time point. The output GPs (one for each gene that might be controlled by the regulator) map the regulator value at a time point to the value of the gene at the same time point. If the relevance parameter of one of these output GPs is low it means that the gene is unlikely to be regulated by the regulator.

In the training phase, both the smoothness (relevance) parameters as well as the hidden values of the regulator need to be estimated. We achieve this by simple maximum a posteriori (MAP) estimation. For longer time series or larger number of regulators, MAP estimation of the hidden values might not work so well any more and alternative methods, such as Markov Chain Monte Carlo methods, need to be considered to sample from the hidden values (Neal (1998) [12]). For comparison we also derive the hidden regulator profile using the NCA algorithm (Liao et al., 2003).

The binding motif of DosR is known. The presence of this motif in the upstream region of a gene is an additional indication that the gene might be regulated by DosR. Consequently, we combine the relevance parameters of a gene with a score for the presence of the DosR binding motif in a logistic regression to improve on the prediction whether a gene is regulated by DosR or not. The results of applying the GP model to the p53 data from the literature and to our gene expression data are presented in the results section. More details about how we used GPs and logistic regression can be found in the methods section.

### Gene expression studies of DosR regulon

Time course microarray data were obtained from *M. tuberculosis *samples cultivated in a chemostat in order to estimate the TFA of the DosR regulator. As one of the most extensively studied regulons in *M. tuberculosis*, DosR plays a major role in mediating hypoxia response in the organism [13-15]. Several studies attempt to identify DosR-regulated genes through comparison of the changes in the gene expression levels in wild type and *dosR *mutant strains [14,15].

There is some discordance between these studies. One has to consider though that the DosR regulon can be stimulated by different environmental stimuli [15]. It is likely that individual environmental perturbations may affect different subsets of genes in the regulon. The batch cultures used in the previous studies provided constantly changing environmental conditions. Consequently, the resulting bacterial population was physiologically heterogeneous with increased intrinsic biological noise in the subsequent microarray analysis. Different normalisation methods and statistical analyses used in the above studies are also likely to contribute to the discrepancy in the identification of DosR-regulated genes.

### Chemostat cultures

In contrast, the use of chemostat cultures produces a well defined and controlled environment for the bacteria. Chemostat cultures are kept under completely controlled conditions and have a constant inflow of defined nutrients and an outflow of waste products that is monitored until steady state is reached before further experiments (here reduction of oxygen) are undertaken. Homogeneity of the culture guarantees that all bacterial cells experience identical conditions that change in just a few defined and controled aspects. The cause-and-effect relationship between the changes of gene expression levels and environmental stimuli can be effectively established [16]. The time course data used in this study comprises nine time points with steadily reduced oxygen supply (for more details see Zhang et al. (2008) [17]). This allows us to measure gene expression trajectories instead of the simple on-and-off signals from two-condition comparisons. It is generally difficult to conduct this type of time course experiments in batch cultures since there it is almost impossible to avoid local fluctuations in environmental conditions and the concentrations of metabolites.

## Results and Discussion

### Estimating the hidden transcription activity profile of p53

The GPDM model was evaluated on the time course microarray data from Barenco et al. (2006) [8] reconstructing the hidden TFA of p53. Barenco et al. (2006) use a differential equation model for the hidden dynamics of the profile of p53 activity which is estimated from the expression profiles of five known p53 target genes. Profiles consist of seven time points with three replicates at each time point.

**Figure 1:**
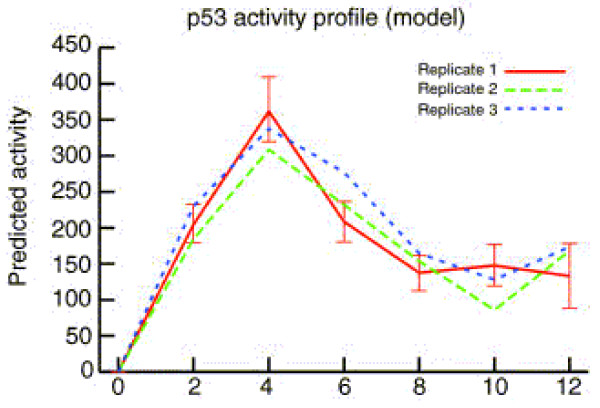
**Activity profile of p53 derived in Barenco et al. (2006) **[8].

**Figure 2:**
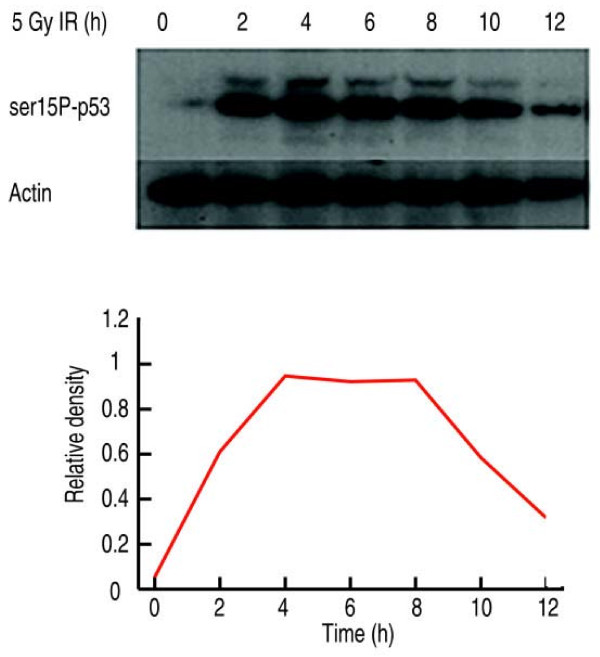
**Experimentally determined p53 activity profile measured by western blot (from Barenco et al. (2006) **[8]**)**.

We applied our GP based dynamic model to the same data as obtained from ArrayExpress (European Bioinformatics Institute). For comparison, the same five genes were used in the GP model and the median of replicates was taken for each gene and log2 transformed for further analysis. Figure 1 displays the estimated TFA from the differential equation model in Barenco et al. (2006) [8]. It can be seen that the predicted p53 activity profile shows a reasonable match to the one determined experimentally by Western blot (figure 2) for the first three time points (4 h), the prediction is less convincing for the rest of the time course (figure 1).

**Figure 3:**
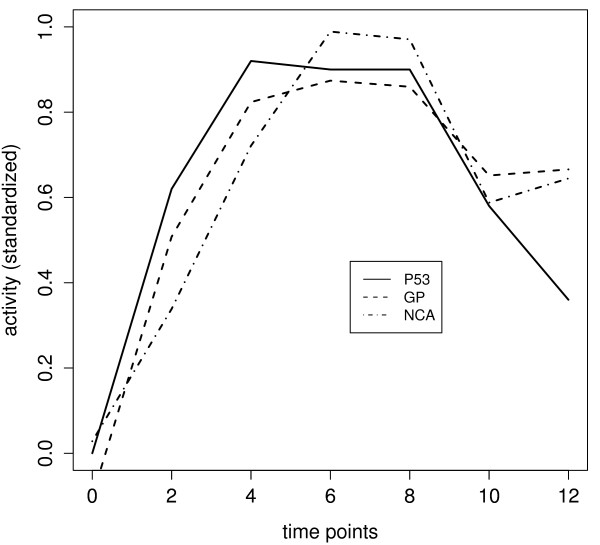
**Activity profile of p53 as estimated by the GP model and the NCA algorithm**.

Figure 3 shows the reconstructed p53 activity derived from the GP model as well as the profile reconstructed according to the NCA algorithm. The GP profile is in better accordance with the experimentally determined profile (figure 2) than the others. Only the last time point (12 h) deviates from the experiment, when the reconstructed TFA stays on the same level as the previous time point (10 h), while the experimentally determined p53 concentration drops off. However, close examination of figure 1 reveals that the band of structure protein actin used as control at 12 h is weaker compared with those at the previous time points. It might be possible that the decline in p53 protein concentration at 12 h (figure 2) is due to an experimental artifact. In any case, table 1 shows the mean of squared differences (SSE) and the correlations between the experimentally determined p53 concentration and the estimated p53 activity profiles from the GP model (GP), the NCA model, and the model used by Barenco et al. (2006). According to these measures the estimated p53 profile from our model is closer to the experimental one than either the NCA or the profiles obtained by Barenco et al.

**Table 1:** Comparison of the estimated p53 activity profile.

	GP	NCA	rep1	rep2	rep3
MSE	0.16	0.26	0.29	0.29	0.24
Corr	0.89	0.85	0.76	0.76	0.81

The table lists the means of squared differences (MSE) after standardization and the correlations between the experimentally determined p53 concentration and the estimated p53 activity profiles from the GP model (GP), the NCA model (NCA) and the model used by Barenco et al (2006). rep1, rep2, and rep3 are the estimations using three replicates, respectively, from Barenco et al.

### Estimating the hidden transcription activity profile of DosR

**Figure 4:**
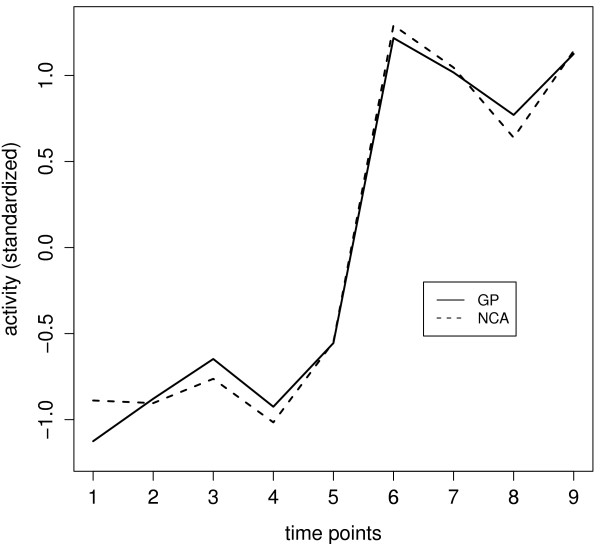
**Activity profile of DosR as estimated by the GP model and the NCA algorithm**.

**Figure 5:**
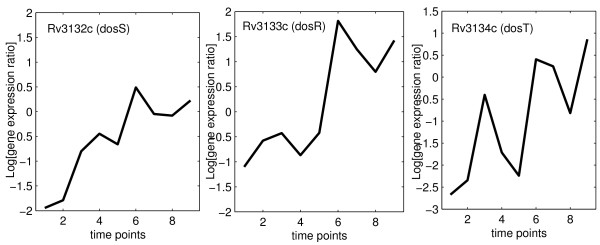
**Expression profiles of three genes in the dosR operon**.

The TFA of DosR was estimated using 21 documented DosR-regulated genes, which also contain the DosR binding motif (table 2) [14]. Figure 4 displays the predicted TFA of DosR according to the GP model as well as the NCA algorithm. The profiles are quite similar, but we will see below that GP relevance scores are much better suited to idenfify dosR dependent genes than the NCA scores. The operon encoding DosR consists of three genes: Rv3134c (*dosT*), Rv3133c (*dosR*), Rv3132c (*dosS*). When compared with the gene profiles of DosR operon (figure 5), it might be seen that the estimated TFA of DosR is strikingly close to the mRNA profile of gene *dosR *itself (Rv3133c) even though *dosR *was not included in the training gene set for estimating its TFA. The close match between the expression pattern of *dosR *and the TFA of DosR can be expected, since the DosR operon has been reported as an auto-regulated operon [18]. However, *dosR *is part of an operon starting with gene Rv3134c, which was used in the training set. Interestingly, there is a noticable discrepancy between the profile of Rv3134c and TFA of DosR (figure 5).

**Table 2:** Ranks of DosR training genes ranked by motif and relevance scores.

ORF	*x*_*motif*_	*x*_*nonLin*_	*x*_*lin*_		
Rv0079	185	13	14	18	15
Rv0569	21	46	38	34	24
Rv0571c	22	12	20	5	7
Rv0574c	83	16	15	6	5
Rv1733c	6	14	1	18	1
Rv1737c	1	5	16	2	6
Rv1738	2	415	389	398	378
Rv1813c	37	7	10	29	4
Rv1997	9	59	82	76	63
Rv2005c	182	9	6	3	3
Rv2006	27	27	24	34	8
Rv2007c	41	6	11	3	3
Rv2031c	8	1	3	1	1
Rv2032	5	2	2	1	1
Rv2626c	10	3	4	1	2
Rv2627c	3	22	9	42	1
Rv2628	4	28	23	24	8
Rv3130c	73	11	7	3	3
Rv3131	49	29	18	20	6
Rv3134c	7	53	41	41	24
Rv3127	78	10	22	3	8

The table shows the rankings of the 21 training genes with respect to different scores: x_*motif *_denotes motif scores, x_*nonLin *_stands for nonlinear scores and x_*lin *_linear scores in the equation (see equation 4).  and  are the nonlinear and linear relevance scores in the leave-one-out cross validation tests which exclude the corresponding genes in the first column from estimating DosR TFA. Note that since each leave-one-out test produces its own ranking, rank numbers are not unique in the last two columns.

### Genome-wide search of DosR-regulated genes

As outlined in the introduction and detailed in the methods section, estimated relevance parameters of the output GP can be used to assess the strength of the influence of the reconstructed TFA of DosR on genes. The profile can also be used to estimate GP parameters for a new gene not involved in the training phase. Two parameters are of interest, one  representing the linear component of the regression of the profile of gene *g *on the TFA, and one  representing the nonlinear component. Essentially, GP training tries to fit a linear regression if possible and adds a nonlinear component only in case there is too much variability unexplained by a linear relationship. We also calculate a motif score  for the presence of the DosR binding motif in the upstream region of gene *g*.

For each of the 2509 operons in the genome of *M. tuberculosis *the first gene was selected to represent the operon (in fact, many of the operons consist of a single gene). Operon definitions were taken from the Biocyc database [19]. Genes were removed from the time course data if the expression level at the last time point (t9) was lower than that at the first time point (t1), since we are only interested in genes which are upregulated by DosR. The relevance scores ,  and motif scores  (see equation 4 in methods) were calculated for the remaining 1008 genes.

**Figure 6:**
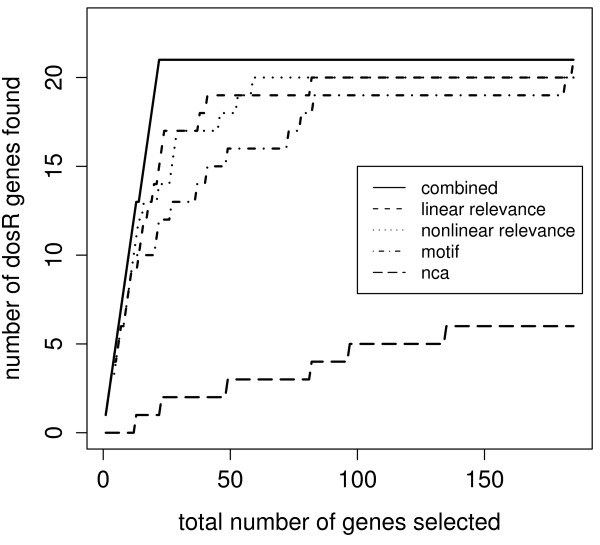
**Ranks of documented DosR-regulated genes among top 185 genes ranked by motif, linear and nonlinear relevance and NCA scores**. The top 4 scorings comprise all 21 DosR-regulated genes within the 185 genes.

The relevance and motif scores can be used separately as a discriminator for ranking genes for regulation by DosR. Figure 6 shows how many of the 21 documented DosR-regulated genes used for training of the dynamic model are among the highest scoring genes. The figure also shows the disappointing performance of scores taken from the NCA. Although the NCA profile is in good agreement with the GP profile (figure 4), the weights linking this profile to the genes are completely unsuitable for discrimination between regulated and nonregulated genes. Table 2 displays the ranks of DosR genes by decreasing relevance and decreasing motif scores.

**Figure 7:**
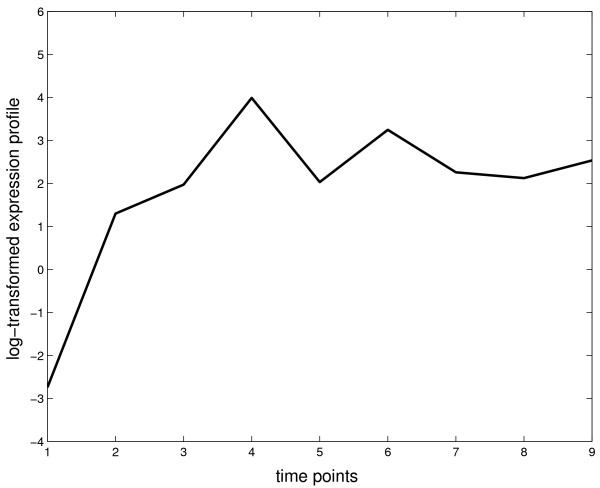
**Log-transformed expression profile of Rv1738**.

Despite having one of the highest motif scores, gene Rv1738 has low ranks in terms of relevance scores, possibly because Rv1738 exhibits a different expression pattern (figure 7) from the rest of DosR-regulated genes in the training set. It was up regulated from time point 2 instead of time point 6 as was the rest of DosR-regulated genes. This is likely due to the high promoter affinity of Rv1738, which may cause saturation of transcription activity as early as at time point 2.

**Figure 8:**
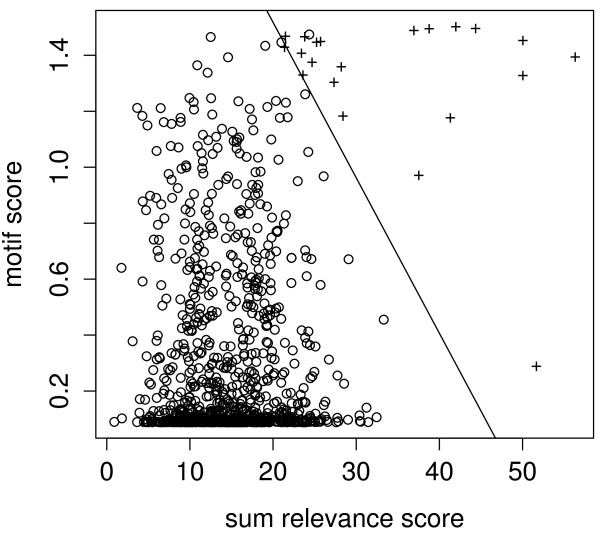
**Plot of motif scores vs relevance scores**.

Figures 8 shows a comparison of the sum of relevance scores derived from the GP model with the motif scores. Figure 6 show that the relevance scores outperform motif scores as a discriminator for ranking DosR-regulated. However, neither is able to identify all the documented DosR-regulated genes. Moreover, they seem to be complementary as discriminators as seen by the clear separation in figure 8. We therefore use a logistic regression of the binary outcome regulated/nonregulated on the relevance score and the motif score (see equation 5 in the methods section) to combine these scores for predicting DosR-regulated genes.

The logistic regression results in a probability *p*_reg _that a gene is regulated by DosR given its scores. As can be seen in figure 8 the separation between DosR regulated genes and the rest is quite clear. This is of course due to the 21 genes being used as training genes in the logistic regression. In order to obtain further genes potentially regulated by DosR looked at the top 34 genes in the ranking of logistic scores. This includes ten novel genes, which we plan to investigated for DosR regulation in future experiments. Three more genes (Rv2623, Rv2625c, Rv2629) in this list have DosR-binding motifs in their upstream region These genes were previously reported as only indirectly regulated by DosR [14].

**Figure 9:**
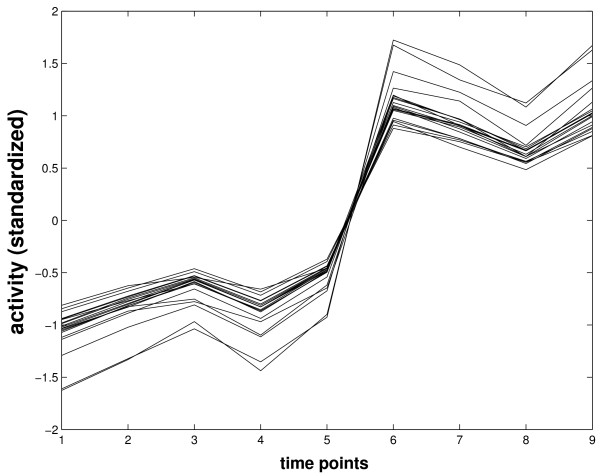
**Estimated TFA of DosR from leave-one-out analyses**.

One objection to the current analysis might be that the 21 DosR-regulated genes were used in training as well as prediction. We therefore repeated the analysis calculating scores for each of the 21 genes in turn using only the remaining 20 genes for training. Figure 9 displays the estimated TFA of DosR in this leave-one-out analyses. We observed that the exclusion of any training DosR-regulated gene had little impact on the estimated TFA. The same was found for predicting novel target genes. In fact, all the leave-one-out analyses resulted in exactly the same rank order among the 21 training genes with respect to the two relevance score and the same predicted DosR-regulated genes as those from the analysis including all the documented DosR-regulated genes in the training data (for a comprehensive list of genes predicted to be DosR regulated see table 3).

**Table 3:** Top ranking potentially DosR-regulated genes.

Rv acc.	description	gene name
Rv0079**	hypothetical protein	
Rv0311*	possible remnant of A transposase	
Rv0482*	UDP-N-acetylenolpyruvoylglucosamine reductase	murB
Rv0569**	hypothetical protein	
Rv0571c**	hypothetical protein	
Rv0574c**	poly-gamma-glutamate synthesis protein	
Rv0654*	putative dioxygenase	
Rv0835*	possible lipoprotein LpqQ	ipqQ
Rv0946c*	glucose-6-phosphate isomerase	pgi
Rv1371*	probable conserved membrane protein	
Rv1733c**	probable conserved transmembrane protein	
Rv1737c**	MFS transporter, NNP family, nitrate transporter	nark2
Rv1738**	hypothetical protein	
Rv1813c**	hypothetical protein	
Rv1954c*	hypothetical protein	
Rv1997**	cation-transporting ATPase	ctpF
Rv1998c*	hypothetical protein	
Rv2005c**	hypothetical protein	
Rv2006**	putative trehalose/maltose hydrolase	otsB1
Rv2007c**	ferredoxin	fdxA
Rv2031c**	heat shock protein hspx	hspX
Rv2032**	Conserved hypothetical protein Acg	acg
Rv2623	hypothetical protein	TB31.7
Rv2625c	probable conserved transmembrane alanine and leucine rich protein	
Rv2626c**	hypothetical protein	
Rv2627c**	hypothetical protein	
Rv2628**	hypothetical protein	
Rv2629	hypothetical protein	
Rv3044*	putative iron (III) dicitrate transport system substrate-binding protein	fecB
Rv3081*	hypothetical protein	
Rv3127**	hypothetical protein	
Rv3130c**	hypothetical protein	
Rv3131**	hypothetical protein	
Rv3134c**	hypothetical protein	

The 34 top ranking genes according to the logistic score. Genes with one * are the novel DosR-regulated genes detected in this study, while those with two * are the documented DosR-regulated genes in the training data. Genes with no * were previously reported as indirectly regulated by DosR, but were found to contain DosR-binding-motifs in this study.

## Conclusions

In this study, we proposed the use of Gaussian process regression for reconstructing the hidden TFAs from time course gene expression data. The proposed approach, in contrast to previous methods for the same purpose, allowed us to model complex dynamics in TFAs and nonlinear and linear interactions between TFs and target genes. Through the application to two experimental data sets we show that the GP model is able to reconstruct the hidden TFAs from time course microarray data reliably.

The *M. tuberculosis *time course microarray data used here have been generated from chemostat cultures, where changes in gene expression levels can be contributed to a single environmental stimulus, in this case, a rapid drop in oxygen tension. This is an advantage over previous DosR regulon studies [13-15] using batch cultures, where uncontrolled local heterogeneity in the conditions of the cultures is a major problem. Using the estimated TFA of DosR, we proceeded to detect DosR-regulated genes in a low oxygen environment. By combining the relevance parameters derived from a GP model and sequence information, we confirmed all the documented DosR regulated genes. By and large the motif scores and relevance scores agree and are larger for DosR-regulated genes. However, there is enough complementary information left in both so that a combination of both scores by a logistic regression improves the classification considerably. This gives confidence that further target genes can be found by ranking genes according to this score. We identified new putative target genes, which will become the focus of our future research.

## Methods

### Time course experiments

*M. tuberculosis *H37Rv was grown in continuous culture to steady state under aerobic conditions (10% DOT) at pH 6.9 and 37*C, in a chemostat, which was controlled by a Brighton Systems controller unit. Cells were grown under carbon-limitation at a dilution rate of 0.03h-1 and a mean generation time of 23 hr. The culture was switched from continuous to batch growth just prior to the start of the time course. The set point on the chemostat controller was reduced from 10% DOT to 0.2% DOT and the oxygen level dropped to the lower set point over 15 minutes. The approach of using continuous culture was adopted in order to generate cells that were growing with the same mean generation time under defined and controlled conditions. The controlled chemostat system was also advantageous as the oxygen level could be monitored throughout the time course. The time point at which each sample was taken was not dictated by the time that had lapsed between each time point (although this was also recorded), but the DOT in the culture (for more details see Zhang et al. (2008) [17]).

Microarray RNA was extracted from cell samples (10 ml) taken at each time point according to the method described previously [16]. Three separate labelling reactions were carried out on each RNA sample, giving three arrays for each time point using the microarray method described previously [16]. In summary, each Cy5-labelled cDNA generated from an RNA sample was co-hybridised with Cy3-labelled DNA generated from *M. tuberculosis *H37Rv genomic DNA.

The resulting gene expression data used in this analysis were log 2 transformed intensity ratios, defined as intensity values of Cy5-labelled cDNA relative to Cy3-labelled DNA. Prior to log 2 transformation, the arrays were preprocessed by the software Bluefuse http://www.cambridgebluegnome.com to estimate signals and subtract background. Then the same normalisation procedure as used in Bacon et al. (2007) [20] was applied to the microarray data to reduce experimental noises.

### Model specification

The model to reconstruct the hidden TFA was built on the Gaussian Process dynamic model (GPDM) [10], which was used to track human motions. The GPDM is augmented with nonlinear latent dynamic variables and a probabilistic mapping from the latent space to the observations. We extended the GPDM further by introducing gene-specific Gaussian processes in the mapping function to model gene-specific regulatory controls. We assume first that the hidden TFA  for TF *i *(*i *= 1, ..., *F*) across time points *t *is given. The activities of all TFs at time point *t *are . The observed gene expression levels for gene *g *are . A state-space model is given by

where ϵ_*x*, *t *_and ϵ_*y*, *t *_are Gaussian noise terms. Θ_*x *_and Θ_*y*, *g *_are the hyperparameters for the Gaussian processes defining *f *and *h*. In our applications the number *F *of transcription factors is one and *x*^*t *^is just a real value. In the following,  are all the hidden and  are all the observed data.

The marginal data likelihood is a multivariate Gaussian density:

where *C*_*g *_is a *T *× *T *covariance matrix. The entries *C*_*g *_(*t*, *s*) of *C*_*g *_are parameterised by a gene specific parameter vector ***β***_*g *_as follows (for example, Williams et al. (1996) [21])(1)

where *s *and *t *are time points and *δ*_*st *_= 1 if *s *= *t *and 0 otherwise. Here we deal with only one hidden regulator (*F *= 1) for simplicity.

The parameters ***β***_*g *_= {*β*_1, *g*_, *β*_2, *g*_, *β*_3, *g*_, *β*_4, *g*_, *β*_5, *g*_} in the covariance function are estimated by maximising the likelihood. The first part, *β*_1, *g*_, in equation 1 is the variance bias and accounts for a constant correlation that exists between any two input points. The second term, *β*_2, *g*_, models the linear interactions between the inputs. This term also adds a nonstationary component to the process. The exponential part reflects the nonlinear dependencies on the inputs where the scale parameter *β*_3, *g *_is the overall strength of nonlinear dependencies and *β*_4, *g *_is the degree to which the input controls the values of outputs. *β*_2, *g *_and *β*_4, *g *_are referred to as relevance parameters in this paper. The last term *β*_5, *g *_represents an additive noise.

Similarily, the distribution of hidden TFA profile *X *is

where *x*^*out *^= (*x*^2^, ..., *x*^*T*^)', *x*^1 ^is the TFA at time point 1 with the Gaussian prior distribution *P *(*x*^1^), and the (*T *- 1) × (*T *- 1) covariance matrix *K *is constructed from *x*^*in *^= (*x*^1^, ..., *x*^*T*-1^)' similar to equation 1 but with the parameters ***α ***= (*α*_1_, *α*_2_, *α*_3_, *α*_4_, *α*_5_):(2)

### Model Learning

The hidden TFA and parameters in the covariance functions were estimated by maximising the likelihood

for some ***α***, ***β***, and *x*. The parameters were optimised in log space with priors of a Normal distribution on their log values:

The hyperparameters {*μ*_*β*_, *τ*_*β*_} are set to encourage sparsity, which is useful for predicting unknown target genes using the relevance parameters *β*_2, *g *_and *β*_4, *g *_given the estimated TFA. The sparse priors amount to an automatic relevance determination (ARD) mechanism embedded in the GP model in that the estimated relevance parameters for genes not under DosR control are encouraged to be close to zero [22]. In this study, we set *μ*_*β *_= *μ*_*α *_= -10 and *τ*_*β *_= *τ*_*α *_= 100. However, in practice we found that the model is robust to different priors used.

Summarising, we look for ***α***, ***β***, and *x *that optimise the function(3)

Having obtained the hidden TFA of DosR, the derived TFA can be used to identify unknown target genes regulated by DosR. In this case, we only need to estimate the relevance parameters *β*_*g *_for gene *g *in question, that is, we only use the parts of the above equation involving *β*_*g*_

### NCA analysis

A suitable version of the NCA algorithm by Liao et al. (2003) [1] can be implemented as follows. If *Y *= () is the matrix of gene expression levels, then we look for a profile vector *p *and a weight vector *a *of gene associations such that the outer product matrix *ap*^*T *^is close to Y as measured by the sum of squared elements of the difference matrix *Y *-*ap*^*T*^. A simple scheme of alternatively regressing the columns of *Y *on *a *and then the rows of *Y *on *p *stabilises on the solution. Using an algorithm such as FastNCA (Chang et al., 2008 [23]) yields the same result.

### Binding motif analysis

Park et al. (2003) [14] discovered the DosR-binding motif, a 20 bp palindromic consensus sequence, by analysing shared sequence motifs upstream of DosR-regulated genes with the motif discovery program YMF. They also provided a scoring matrix *S*_*ij *_with entries that are the log-likelihood ratios of each base at each position [14]. The scoring matrix *S*_*ij *_is used to calculate the log-likelihood of a putative DosR-binding motif to improve the accuracy of the prediction of novel target genes in addition to gene expression data.

Sequences (300 bp) upstream of the translation start site of the first gene in each operon were extracted to search against the DosR-binding motif. Then for each gene the final motif score *x*_*motif *_is the sum of all the positive motif scores from the corresponding upstream sequences:

where *J *is the length of the motif and *K *is the number of motifs with positive scores, i.e.,  obtained from the upstream sequence of a gene. No overlapping motifs are allowed.

The logistic regression model provided a weighted combination of the motif scores and the relevance scores derived from the GP model. There are 21 genes documented to be directly regulated genes by DosR [14]. These genes and an equal number of randomly selected genes without any reported *dosR *association were used for the training of the logistic regression model as positive and negative training datasets respectively:(4)

where *p*_reg_(*g*) denotes the probability that a gene *g *is regulated by DosR. The relevance score  denotes the linear relevance parameter for gene *g*, i.e., *β*_2, *g*_,  stands for the nonlinear relevance parameter *β*_4, *g *_(for *β*_2, *g *_and *β*_4, *g *_see equation (1)) and  is the DosR-binding motif score as described above.

The estimated weights *w*_1 _and *w*_2 _were then used to compute the probability of any gene *g *being regulated by DosR:(5)

## Authors' contributions

YZ and LW developed the statistical approach and YZ carried out the data analysis. JB and KAH carried out the chemostat culture and microarray experiments. YZ, JB, and LW contributed equally to the manuscript. All authors read and approved the final manuscript.
